# Propionic Acidemia: Gray Matter Disease Meets Subcortical Leukodystrophy

**DOI:** 10.1002/jimd.70101

**Published:** 2025-11-04

**Authors:** Hannah Fels‐Palesandro, Friederike Hörster, Dorothea Haas, Florian Gleich, Sven F. Garbade, Stefan Kölker, Inga Harting

**Affiliations:** ^1^ Department of Neuroradiology University Hospital Heidelberg Heidelberg Germany; ^2^ Medical Faculty University Hospital Heidelberg, Center for Child and Adolescent Medicine, Division of Pediatric Neurology and Metabolic Medicine Heidelberg Germany

**Keywords:** basal ganglia, cerebellar watershed, leukodystrophy, leukoencephalopathy, metabolic stroke, MRI, propionic acidemia

## Abstract

Imaging literature on propionic acidemia (PA) is predominantly concerned with deep gray matter changes. In order to investigate the spectrum and patterns of MRI changes, 45 MRI scans of 13 patients (0.31–33.2 years) were systematically analyzed. Deep and cortical gray matter changes were associated with acute metabolic decompensation. Striatum was affected in 10/13; T2‐hyperintensity was often mild, normalizing and becoming T2‐hypointense in one patient each without movement disorder. Pallidum was T2‐hyperintense in the 3 infants imaged for acute decompensation, with restricted diffusion in 2/3 and normalizing in 2/2 with follow‐up. Dentate and thalamus were T2‐hyperintense in 7/13 and 2/13, respectively, without resolution. Cerebellar watershed injury (4/13) and cortico‐subcortical involvement with transiently restricted diffusion, T2/FLAIR‐hyperintensity, and/or swelling (3/13) were more common with seizures during decompensation. A pattern of predominantly subcortical leukoencephalopathy was observed in 7/9 patients aged 9 years and above. Small foci of restricted diffusion, T2/FLAIR‐hyperintensity, and variable T1‐hypointensity evolved into bilateral, sometimes extensive changes with restricted and facilitated diffusion, decreasing in 3/6 with follow‐up MRI at 14.7–19.5 years. Volume deficit was common, including a thin brainstem in 9/13. Predominantly subcortical white matter changes are a characteristic finding in older children with PA, might reflect microvacuolation, and may improve. Transient pallidal changes in infants with acute decompensation might represent myelin splitting during active myelination of white matter‐rich pallidum. Cortico‐subcortical changes and cerebellar watershed injury during acute metabolic decompensation were more likely with seizures; like the more common striatal involvement they occurred without age‐predilection.

## 1 Introduction

Propionic acidemia (PA; MIM #606054) is a rare, autosomal recessive metabolic disease estimated to occur in 1:50000–1:500000 births [1]. It is caused by a deficiency of propionyl‐CoA carboxylase (PCC) due to biallelic pathogenic variants in *PCCA* or *PCCB*. PCC catalyzes the formation of methylmalonyl‐CoA from propionyl‐CoA. It is a key enzyme in the anaplerotic metabolic pathway that channels succinyl‐CoA to the tricarboxylic acid (TCA) cycle via degradation of branched‐chain amino acids, odd‐numbered long‐chain fatty acids, and side chains of cholesterol and via propiogenic gut bacteria [1, 2, 3].

In PA, accumulating propionyl‐CoA induces lactic acidosis and hyperammonemia by inhibition of the pyruvate dehydrogenase complex and N‐acetylglutamate synthetase, respectively. Accumulating 2‐methylcitrate, a compound with multiple inhibitory effects on the TCA cycle, further aggravates the bioenergetic impairment in PA [4]. Accumulating propionate, among other effects, inhibits Na+/K+‐ATPase, GABA transaminase, and induces oxidative stress [5, 6, 7]. Acute metabolic decompensation is triggered by catabolic stress, e.g., intercurrent infection or prolonged fasting, when protein degradation acutely increases circulating toxic metabolites with resultant metabolic acidosis and hyperammonemia. As patients become symptomatic despite good metabolic control, the resulting multisystem disorder is best described as sequelae of acute decompensations of varying severity and also of chronic, accumulating neurotoxicity [5].

PA is usually diagnosed in early childhood with a neonatal or a late‐onset form [1, 8]. Without sufficient treatment, metabolic decompensation with vomiting and lethargy may evolve to coma and death [1, 9]. Seizures, muscular hypotonia, and movement disorder may be associated or develop independently in up to 53%, 100%, and 40%, respectively [1]. Developmental delay and cognitive disability as complications of chronic neurotoxicity are reported in 59%–100% of patients [1], while optic neuropathy in adolescents is less frequent and neuropsychiatric symptoms are rare complications [1, 3, 5]. Cardiomyopathy is the most severe somatic complication (39% in a single‐center study) [10]. Chronic renal dysfunction sometimes develops in older patients [1, 8]. Hepatic involvement with elevation of AFP to liver cirrhosis has also been described [11].

MRI changes in PA have to date been reported in approximately 130 patients, including numerous cases and case series. Reports, however, have predominantly focused on deep gray matter (GM), particularly striatal (putamen and caudate) changes [11, 12, 13, 14, 15, 16, 17, 18, 19, 20, 21, 22, 23, 24, 25, 26, 27, 28, 29, 30, 31, 32, 33, 34, 35, 36, 37]. Reported changes further include cortical edema [19, 22, 23, 24, 29, 38], myelination delay [12, 15, 20, 28, 33], white matter (WM) abnormalities [33, 36], volume deficit [12, 13, 14, 15, 16, 20, 23, 24, 28, 39, 40, 41, 42], and cerebellar hemorrhage [37]. Moreover, cohorts published subsequently to the first group of 15 patients with MRI [12] were clinically focused and/or have dealt more summarily with imaging [20, 25, 28, 36]. In order to investigate the spectrum and pattern of MRI changes in PA, we systematically analyzed 45 MRI scans from 13 individuals, the largest MRI cohort so far.

## 2 Patients and Methods

Individuals with confirmed PA and available brain MRI studies in DICOM format were recruited through the E‐IMD database (https://www.eimd‐registry.org/), a multicentre observational study on intoxication‐type inherited metabolic diseases. All procedures were in accordance with the ethical standards of the responsible committee on human experimentation (institutional and national) and with the Helsinki Declaration of 1975, as revised in 2024. Informed consent to participate in the study was obtained from all individuals, their parents, or legal guardians. The study was approved by the ethics committee of the University Hospital Heidelberg (S‐525/2010).

Clinical, biochemical, and genetic data were retrieved from the E‐IMD database and individual chart review, including neurological findings, cardiac, renal, and hepatic involvement, plasma ammonia, onset of symptoms, and mode of diagnosis. As patients were seen at different centers, availability and depth of clinical information varied. Indication for MRI was categorized as acute for onset of acute metabolic decompensation within the last 14 days, seizures without metabolic decompensation, follow‐up without new clinical manifestation, and other indications, e.g., trauma or movement disorder.

MRIs, obtained for clinical indications between 9/1997 and 6/2024 at different centers, were systematically and independently reviewed by IH and HFP, blinded for clinical and biochemical findings, with a second review and final agreement performed during consensus meetings. T2‐weighted and T1‐weighted images were available for all patients and MRIs, diffusion‐weighted imaging (DWI) with apparent diffusion coefficient (ADC) for 11/13 individuals (36/45 MRI). MRI was assessed for the presence and extent of signal changes of GM and WM. Diffusion was assessed as restricted only if there was corresponding hypointensity on ADC maps. Myelination was assessed visually using milestones [43, 44] and a myelination score [45]. T2‐gradient‐echo or susceptibility‐weighted images (SWI; 7 patients/16 MRI) were checked for hypointensity due to calcifications and/or blood degradation products. Atrophy was visually evaluated as widened cerebrospinal fluid (CSF) spaces, thin corpus callosum, and/or brainstem for age. Bicaudate ratio (BCR), defined as the minimum intercaudate distance divided by the transverse width of the inner table of the skull at the same level, i.e., the outer surface of CSF, was used as a surrogate parameter of supratentorial brain atrophy. As BCR varies with age, *z*‐scores were calculated using age‐adapted control values [46]. The intracranial optic nerve was visually rated as thin or normal compared to age‐matched subjects with normal imaging. Descriptive statistics used to summarize patient characteristics and *z*‐scores of BCR were calculated using R version 4.5.0 [47].

## 3 Results

### 3.1 Study Cohort and Clinical Phenotype

A total of 13 patients were identified and evaluated in the study (6/13 female; Table S1). Diagnosis was based on the typical pattern of organic acids in urine and subsequently confirmed by molecular genetic analysis in 9/13. Eleven patients had early onset as neonates with selective metabolic work‐up. 2/13 were identified by a newborn screening pilot study, but became symptomatic at 3.7 and 5.1 months despite early treatment. All patients received long‐term treatment with a low‐protein diet, precursor‐free amino acid supplementation, and l‐carnitine and emergency treatment during acute metabolic decompensation according to guidelines [1, 3]. Carglumic acid was used in p1 and p5 without significant improvement of ongoing metabolic decompensation. Five early‐onset patients died at 15.8–20.2 years, four in association with severe metabolic decompensation and one due to cardiomyopathy.

MRI was obtained as part of clinical care with age at first MRI 0.3–17.7 years (median 10.7) and 1–8 available follow‐up MRI after 3 days to 15.5 years in 11/13 patients (Table 1). The most common indications for MRI were acute metabolic decompensation (15 MRI, 9 patients) and follow‐up without new manifestations (6 MRI, 6 patients, 2/6 within 2 months after decompensation), followed by seizures without acute decompensation (4 MRI, 3 patients), fatigue and vomiting (2 MRI, 1 patient), movement disorder, progressive gait disturbance, visual impairment, new optic atrophy, sensorineural hearing loss, trauma, suspected cholesteatoma, and psychosis (1 MRI). Pilocytic astrocytoma in a patient with seizures was followed for 6.3 years with 7 pre‐ and postoperative scans. No information on indication was available for 3/4 MRI of deceased patient p11.

**TABLE 1:** MRI changes.

PID	MRI								Deep gray matter^a^							Cortex	Infratent			Atrophy	White matter		
	FU	Age (yrs)	NH_3_ at MRI (μmol/L)	Indication	If acute: day of decomp.	If acute: max. NH_3_ until MRI (μmol/L)	Decomp. before FU0	Mov. disorder	Put.	Caud.	Pall.	Thal.	Dent.	Restr. diff. (ADC)	T1‐hyperint.	Supratent.	Cerebellar watershed injury	Cerebell. atr.^b^	Thin brainstem	Supratent.	Delayed/incompl. myel.	White matter signal changes (restr./isoint./fac. diff. refers to signal on ADC maps denoting restricted/unchanged/facilitated diffusion)	Thin optic nerve
p1	FU0	0.31	368	Acute	d2	452	n	n	y	y	y	y	n	Pall.	n	y	n	n	y	n	n	Cort.‐subcort. (restr. diff.)	n
	FU1	0.31	86	Acute	d5			y	y	y	y	y	n	Pall.	n	y	n	n	y	n	n	Cort.‐subcort. (restr. diff.)	n
p2	FU0	0.44	170	Acute	d4	170	n	n	y	y	y	n	y	Pall.	n	n	n	n	n	y	y	n	n
	FU1	0.75	70	FU				y	y	y	n	n	y	n	n	n	n	n	y	y	y	n	n
p3	FU0	1.06	72	FU			Neonat.	n	n	n	n	n	y	NA	n	n	n	n	y	n	n	n	n
p4	FU0	1.14	76	Acute	d8	122	Neonat.	n	y	n	y	n	n	n	n	n	y (DWI, unilat.)	n	n	y	y	n	n
	FU1	1.86	80	Acute	d1	180		n	y	n	n	n	n	n	n	y	y (DWI, bilat.)	y	y	y	n	n	n
	FU2	1.89	62	FU	d13			n	y	n	n	n	n	n	n	y	y (DWI, bilat.)	y	y	y	n	n	n
	FU3	12.11	69	Acute	d2	131		n	y	y	n	n	n	n	n	n	y (atr., new DWI adjacent)	y	y	y	n	C.sem. (isoint. diff.)	n
	FU4	12.60	36	Clin. susp. cholesteatoma				n	n	n	n	n	n	n	n	n	y (atr.)	y	y	y	n	C.sem. (isoint. diff.)	n
	FU5	13.22	71	Acute	d5	150		n	n	n	n	n	n	n	n	n	y (atr.)	y	y	y	n	C.sem. (isoint. diff.)	n
p5	FU0	1.22	Unkn.	Seizures			Neonat.	n	y	y	n	n	n	NA	n	n	n	n	y	y	y	n	n
	FU1	2.84	51	Acute	d4	97		y	y	y	n	n	y	n	n	y	y (bilat., FLAIR)	n	y	y	y	Cort.‐subcort., new (restr. diff.)	n
	FU2	2.98	99	FU				Unkn.	y	y	n	n	y	n	n	y	y (min. decr.)	n	y	y	y	Cort.‐subcort., residual (fac. diff.)	n
	FU3	3.35	92	Trauma				Unkn.	y	y	n	n	y	n	n	n	y (bilat.)	n	y	y	y	Cort.‐subcort., residual (fac. diff.)	n
	FU4	11.34	unkn.	Seizures				Unkn.	y	y	n	n	y	n	n	n	y (resid. FLAIR, atr.)	n	y	y	y	Subcort., new (restr. diff.)	n
	FU5	13.83	Unkn.	Visual impairment				y	y	y	n	n	y	n	n	n	y (resid. FLAIR, atr.)	n	y	y	y	Subcort., incr. (restr., old isoint./ fac. diff.)	n
p6	FU0	2.52	Unkn.	FU			Neonat., mult. first yrs.	n	n	n	n	n	y	NA	n	n	n	n	n	n	y	n	y
p7	FU0	3.58	47	Seizures			Mult. (no details)	y	y	n	n	n	n	NA	n	n	n	n	n	y	y	C.sem. (no ADC)	n
	FU1	15.11	120	Acute	d1	120		y	y	y	n	n	n	n	y	n	n	y	y	y	y	Subcort., new (restr., majority fac. diff.) & C.sem.	y
p8	FU0	5.58	69	Movement disorder			At age 6.5 mo	y	y	n	n	n	y	n	n	n	n	n	n	y	y	n	y
	FU1	8.15	62	Seizures				y	y	n	n	n	y	NA	n	n	n	n	n	y	y	n	y
p8	FU2	8.39	33	Pilocytic astr., preoperative				y	y	n	n	n	y	n	n	n	n	n	n	y	y	n	y
	FU3	8.66	66	Pilocyt., postop				y	y	n	n	n	y	n	n	n	n	n	n	y	y	n	y
	FU4	9.17	52	Pilocyt., postop				y	y	n	n	n	y	n	n	n	n	n	n	y	y	n	y
	FU5	10.03	17	Pilocyt., postop				y	y	n	n	n	y	n	n	n	n	n	n	y	y	n	y
	FU6	11.66	Unkn.	Pilocyt., postop				y	y	n	n	n	y	n	n	n	n	n	n	y	y	n	y
	FU7	13.54	38	Pilocyt., postop				y	y	n	n	n	y	n	n	n	n	n	n	y	y	Subcort., new (fac. diff.)	y
	FU8	14.71	Unkn.	Pilocyt., postop				y	y	n	n	n	y	n	n	n	n	n	n	y	y	Subcort, decr. (fac. diff.)	y
p9	FU0	9.03	74	FU			At age 6 mo	Unkn.	n	n	n	n	n	NA	n	n	n	y	y	y	n	Subcort. (no DWI)	n
	FU1	11.59	97	Acute	d2	129		n	y	y	n	n	n	n	n	n	n	y	y	y	n	Subcort., incr. (fac. diff.)	n
	FU2	18.05	50	Acute	d14	120		n	y	y	n	n	y	n	y	n	n	y	y	y	n	Subcort., decr. (isoint./ fac. diff.)	n
p10	FU0	9.77	126	Acute		174	Neonat., mult. first yrs.	Unkn.	y	y	n	n	n	NA	n	n	y (atr.)	y	n	y	y	Subcort. (no DWI)	n
	FU1	10.70	78	Gait disturbance				y	y	y	n	n	n	n	n	n	y (atr.)	y	n	y	y	Subcort., incr. (restr., old isoint./ fac. diff.)	n
p11	FU0	15.40	Unkn.	Unkn.			Multiple (no details)	n	y	y	n	n	y	n	n	n	y (bilat., FLAIR; add. focal atrophy)	n	NA	y	n	n	n
	FU1	17.97	Unkn.	Unkn.				n	y	y	n	n	y	n	n	n	y (atr.)	n	n	y	n	Subcort., new (isoint. diff.)	n
	FU2	18.31	Unkn.	Unkn.				Unkn.	y	y	n	n	y	n	n	n	y (atr.)	n	n	y	n	Subcort., incr. (restr. & fac./ isoint. diff.)	n
	FU3	19.47	Unkn.	Acute	Unkn.	Unkn.		Unkn.	y	y	n	n	y	n	n	n	y (atr.)	n	n	y	n	Subcort., decr. (fac. diff.)	n
p12	FU0	15.81	100	Optic atrophy			Neonat., mult. first yrs.	n	y	y	n	y	n	n	n	n	n	n	y	n	y	n	y
	FU1	23.63	95	Acute	d6	178		n	n^c^, ^a^	n	n	y	n	n	n	n	n	y	y	y	y	Subcort., new (pred. restr. diff.)	y
	FU2	23.77	109	Acute	d2	124		n	n^c^, ^a^	n	n	y	n	n	n	n	n	y	y	y	y	Subcort., incr. (restr., some pre‐existing fac. diff.)	y
p13	FU0	17.67	36	Fatigue, vomiting			Neonat.	n	n	n	n	n	n	NA	n	n	n	n	y	n	y	n	n
	FU1	22.94	62	Psychosis				n	n	n	n	n	n	n	n	n	n	n	y	n	y	n	n
	FU2	23.47	57	Fatigue, vomiting				n	n	n	n	n	n	n	n	n	n	n	y	n	y	n	n
	FU3	33.17	Unkn.	Sensorineural hearing loss				n	n	n	n	n	n	NA	n	n	n	n	y	n	y	n	n

Abbreviations: atr., atrophy; caud, caudate nucleus; C.sem., Centrum semiovale; decomp., decompensation; decr./incr., decreasing/increasing; dent, dentate nucleus;; dev., development; FU, follow‐up MRI; incompl.myel., incomplete myelination; mo, months; mov., movements; musc., muscular; pall, globus pallidum; progr., progressive; put, putamen; restr./isoint./fac. diff., restricted/normal/facilitated diffusion on ADC‐maps compared to normal white matter; (sub)cort., (sub)cortical; supra/infratent., supra/infratentorial; thal, thalamus; yrs., years.

^a^ T2‐hyperintensity unless otherwise stated.

^b^ Not restricted to watershed areas.

^c^ Relat. T2‐hypoint.

At least one episode of acute metabolic decompensation was documented and/or led to an MRI in all patients. Nine patients (15/45 MRI, 0.3–23.8 years) were imaged on days 1–14 during a total of 14 acute episodes, including p1 with a 3‐day follow‐up. Serum ammonia, available for 13/14 episodes, was markedly elevated with a median of 150 μmol/L (range 120–452; reference 12–53 μmol/L, ≤ 110 μmol/L in neonates). In p7 at FU1 and p9 at FU2 with acute decompensation and septic shock, there was predominant and severe lactic acidosis, but only moderately elevated ammonia.

Movement disorder (available information in 13/13 patients, 39/45 MRI) was present in 6/13 patients, all of these with acute metabolic decompensation. 6/13 patients had seizures at the time of MRI, occurring in the setting of acute decompensation in 4/6 (6/10 MRI). All patients except the youngest at 0.3 years had developmental delay, mental retardation, and/or intellectual disability at the time of MRI. Optic atrophy on fundoscopy was documented for 3 patients. Somatic involvement was documented in 8/13 patients, namely cardiomyopathy and/or long QTc interval in 7/13, chronic kidney disease in 2/13 with additional chronic hepatic dysfunction in 1/2.

### 3.2 MRI Changes

The most prevalent MRI change was deep GM involvement in 12/13 patients, followed by WM signal changes, delayed or incomplete myelination, and supratentorial atrophy in 9/13. Injury in cerebellar watershed areas was present in 4/13 and supratentorial cortex was affected in 3/13 patients (Table 1).

#### 3.2.1 Deep Gray Matter Involvement

Deep GM involvement was bilateral and symmetric and most common in basal ganglia (10/13).

**FIGURE 1:**
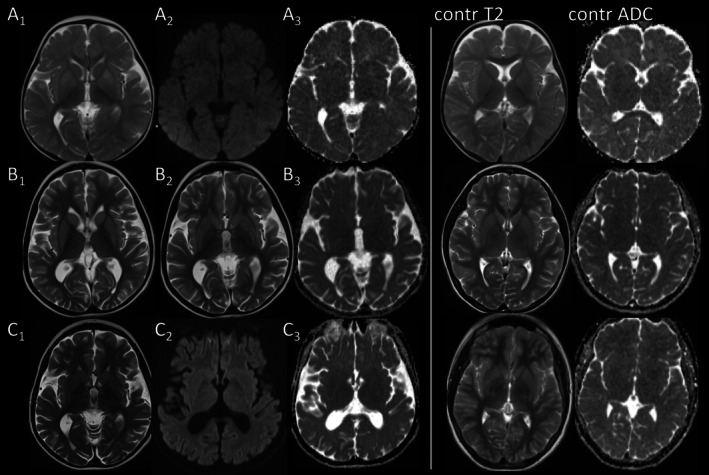
Striatal and reversible pallidal involvement in p4. (A) Swelling and T2‐hyperintensity of putamen and pallidum in p4 at 1.1 years on day 8 of acute metabolic decompensation, but without restricted diffusion. (B) At 12,1 years (FU3) there is new caudate involvement, again without restricted diffusion on day 1 of acute metabolic decompensation with impaired consciousness. Pallidal T2‐hyperintensity had already resolved at prior MRI at 1.9 years (not shown). (C) At 13.2 years (FU5) signal of basal ganglia has normalized with signal of striatum slightly less than that of insular cortex (compare with age‐matched control). Diffusion remains normal, generalized atrophy has increased. (T2w: A_1_–C_1_, B_2_, DWI: A_2_, C_2_, ADC‐maps: A_3_–C_3_.)

**FIGURE 2:**
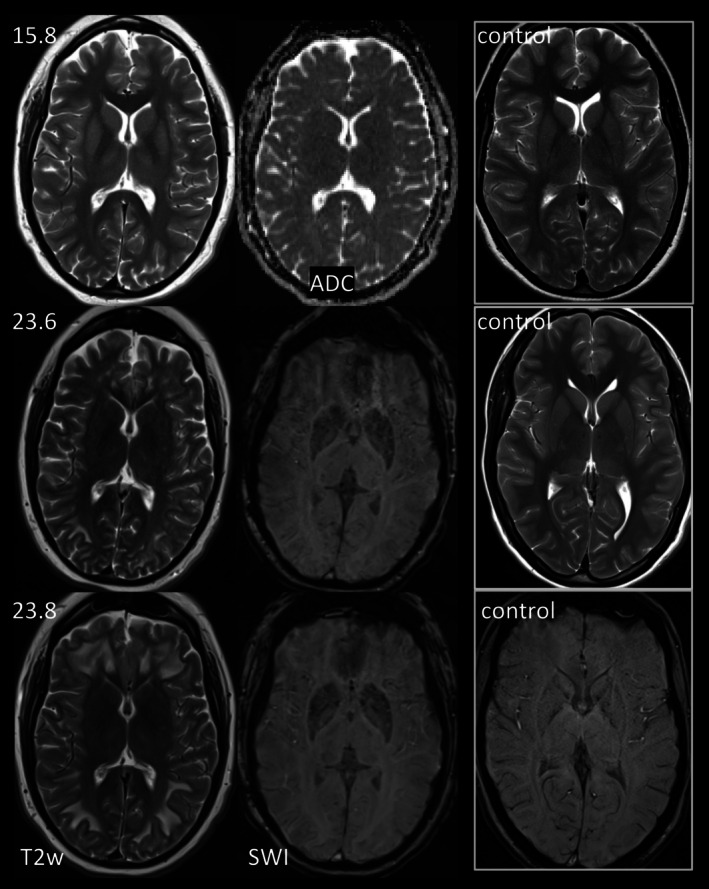
Basal ganglia changes with evolving T2‐hypointensity in p12. At 15.8 years, putamen, caudate nucleus and anterolateral thalamus are T2‐hyperintense in p12 with multiple neonatal acute metabolic decompensations, impaired fine motor skills and intellectual disability; diffusion is normal. By follow‐up at 23.6 and 23.8 years, on day 6 and day 2 of two episodes of acute metabolic decompensation, T2‐hyperintensity has been replaced by mild T2‐ and SWI‐hypointensity.

Striatal T2‐hyperintensity always involved the putamen (10/10), in 9/10 also the caudate nucleus, and without apparent age predilection at 0.31–23.8 years. All patients with striatal involvement had more than 1 MRI. Striatal T2‐hyperintensity was present at the first MRI in 9/10, at FU1 in p9 (0.3–17.7 years). It resolved despite multiple acute episodes in p4 (Figure 1) and in p12 evolved to T2‐ and SWI‐hypointensity by 23 years (Figure 2).

**FIGURE 3:**
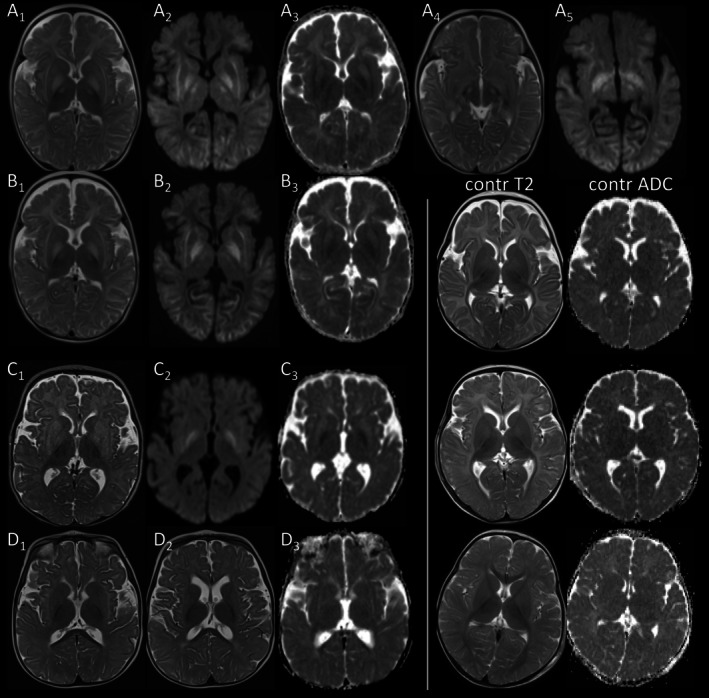
Basal ganglia changes in younger patients. (A, B) T2‐hyperintense pallidum, anterolateral thalamus, and subthalamic nucleus with restricted diffusion in p1 at 0.3 years (A) on day 2 of first acute metabolic decompensation with. Putamen and caudate nuclei are less T2‐hyperintense with facilitated diffusion. (B) Repeat MRT after 3 days (having become comatose) reveals no significant change, in particular no restricted diffusion in striatum. (C, D) T2‐hyperintense putamen and pallidum with restricted diffusion of pallidum on day 4 of first acute metabolic decompensation in p2 at 0.4 years (C). T2‐hyperintensity and restricted diffusion of pallidum have normalized by follow‐up at 0.7 years (D). There is residual, faint, mottled T2‐hyperintensity of putamen (compare with signal of age‐matched control). (T2w: A_1_–D_1_, A_4_, D_2_, DWI: A_2_, A_5_, B_2_–D_2_, ADC‐maps: A_3_–D_3_.)

T2‐hyperintensity of the pallidum was present in three infants imaged during acute metabolic decompensation before the age of 14 months, involving both internal and external portions of the pallidum, and always in conjunction with striatal involvement. In p1 it was unchanged after 3 days, while it had resolved in p2 and p5 on follow‐up after 3.5 and 8.5 months (Figures 1 and 3).

Anterolateral thalamus was involved in 2 patients, namely the youngest and the oldest at 0.3 and 15.8–23.8 years, with concurrent striatal involvement and without resolution on follow‐up.

Dentate nucleus was T2‐hyperintense in 7 patients imaged at 5.2 months to 19.5 years, associated with striatal involvement in 5/7 and the only deep GM involvement in 2/7.

DWI was available for 11 patients, including 14 MRI studies obtained during 13 acute episodes of 8 patients. Timing of MRI was on day 1–5 of admission for 9/13 episodes, until day 14 for 3/13, and unknown for 1/13. In deep GM, restricted diffusion was only observed in the pallidum of the two youngest infants with acute decompensation, in p1 with restricted diffusion also in the thalamus and subthalamic nucleus (Figure 3). No patient had restricted diffusion of the striatum.

#### 3.2.2 Movement Disorder and Deep Gray Matter Involvement

Movement disorder was present in 6/12 patients with available information and confined to patients with MRI‐visible striatal involvement. These included all three patients with volume deficit of basal ganglia (p5, p7, p8; Figure S1). Interestingly, three patients did not develop movement disorder despite 6–11 years' MRI‐visible striatal T2‐hyperintensity, including p4 with resolution and p12 with conversion to T2‐hypointensity.

#### 3.2.3 Cerebellar Watershed Injury

**FIGURE 4:**
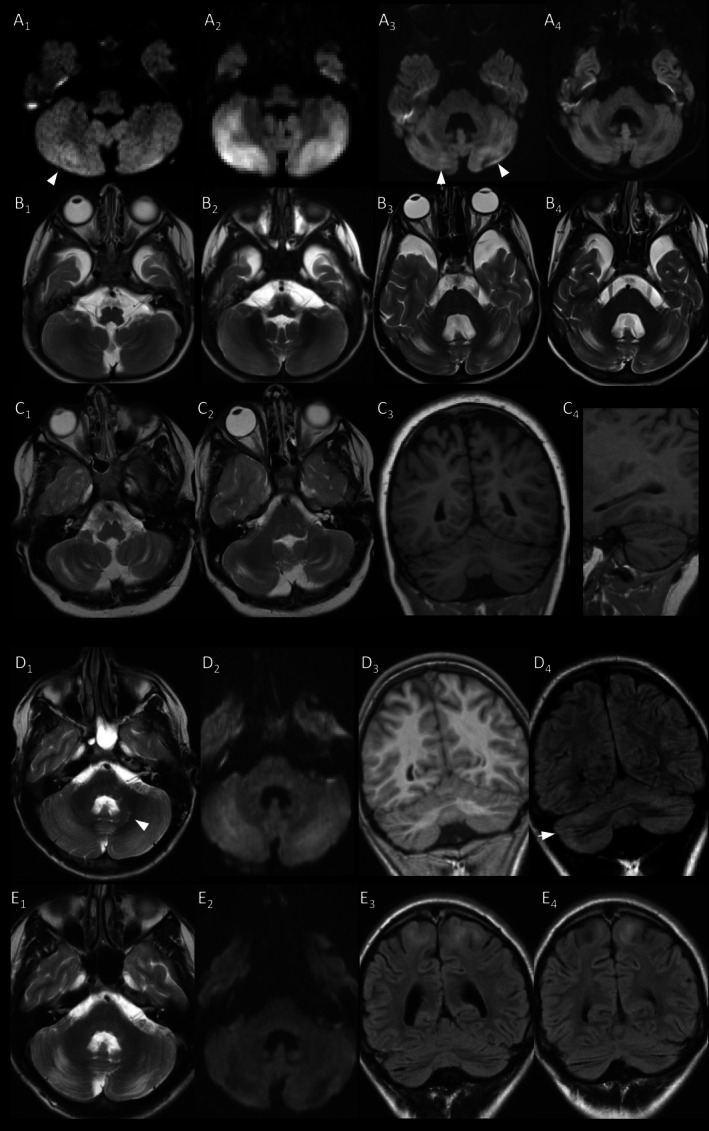
Examples of cerebellar watershed injury. (A, B) Evolution of cerebellar watershed injury in p4 with mild, focal hyperintensity in the right cerebellar hemisphere on diffusion‐weighted image (A_1_) at 1.1 years during acute metabolic decompensation. Hyperintensity is bilateral and more extensive at 1.9 years during another acute episode (A_2_), while T2‐hyperintensity is difficult to appreciate (B_2_). Atrophy with widening of depth of cerebellar fissures has developed by 12.1 years a further acute episode (B_3_). Note the small adjacent areas of increased signal on DWI consistent with acute injury (A_3_), which have resolved on follow‐up at 12.6 years (A_4_; compare with signal of temporal cortex). (C) Atrophy of cerebellar watershed areas in p10 at first MRI at 9.8 years during acute metabolic decompensation with characteristic involvement of depth of cerebellar fissures. (D, E) Combination of focal atrophy consistent with pre‐existing injury (D_4_, arrowhead) and more extensive (sub)acute cerebellar watershed injury with T2/FLAIR/DWI‐hyperintensity and mild T1‐hypointensity in p11 at first MRI at 15.4 years. NB T2‐hyperintense signal of dentate nucleus (arrowhead in D_1_). On follow‐up at 18 years signal changes have been superseded by atrophy. NB new changes of subcortical cerebral WM (E_3,4_), see also Figure 6. (DWI: A_1–4_, D_2_, E_2_; T2w: B_1–4_, C_1,2_, D_1_, E_1_; T1w: C_3–4_, D_3_; FLAIR: D_4_, E_3,4_.)

Four patients had injury of arterial watershed areas of the cerebellum without apparent age‐predilection at 1.1–19.5 years, manifesting as hyperintensity on T2w, FLAIR, DWI, and/or focal atrophy (Figure 4), which was usually bilateral and symmetric. As signal changes were most conspicuous on DWI, cerebellar watershed injury might have escaped detection in the 9 MRI without DWI, including 1/15 MRI for acute metabolic decompensation. DWI and information on MR‐indication were available for 11 patients, of whom 8 were imaged during 13 acute episodes. Acute cerebellar watershed injury, namely new signal changes and/or restricted diffusion, was present in 4/13 acute episodes. Information on neurology was available for 12/13 acute episodes. Acute cerebellar watershed injury was present in 4/5 acute episodes with seizures, but in 0/7 acute episodes without and thus overrepresented in acute episodes with seizures.

#### 3.2.4 Supratentorial Cortex Involvement

Restricted diffusion, T2/FLAIR‐hyperintensity, and/or swelling of supratentorial cortex was present in 3/9 patients imaged during acute metabolic decompensation (3/14 episodes) with involvement of adjacent WM in 2/3. It was extensive and symmetrical in p1, focal and asymmetrical in p4 and p5. On follow‐up, changes were still visible after 3 days, 0.4 and 1.7 months, subsequently resolving in the 2 patients with longer follow‐up.

Regarding seizures as a potential contributing factor, seizures were documented for 6/14 acute episodes. Cortex was affected in 2/6 episodes with seizures compared to 1/8 episodes without seizures. Of note, cortical involvement was not present in three patients (5 MRI) imaged for seizures without associated acute metabolic decompensation.

#### 3.2.5 White Matter Changes

Supratentorial WM involvement was present in 9 patients with 3 different, potentially overlapping patterns, including above‐mentioned cortico‐subcortical WM changes during acute metabolic decompensation in 2/9. A second, infrequent pattern consisted of symmetrical, mildly T2‐hyperintense centrum semiovale and/or corona radiata without restricted diffusion in 2 patients at 3.6–15.1 years.

A third pattern of predominantly subcortical WM involvement was observed in 7 patients at 9–23 years, with a prevalence of 77.8% among the 9 patients first imaged after the age of 5 years. They were already present at first MRI in 2/7 at 9.0 and 9.8 years and in 5/7 were first observed between 11.4 and 23.6 years. Except for p9 with only frontal lesions, all lobes were involved with a frontoparietal predominance, including the central region.

**FIGURE 5:**
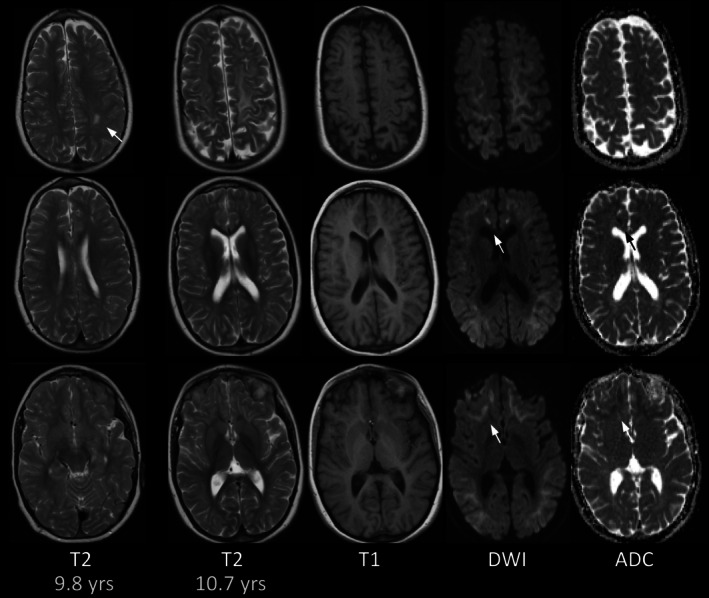
Exemplary white matter changes in p10. At 9.8 years during acute metabolic decompensation there is focal, non‐specific T2‐hyperintensity in left parietal lobe. On follow‐up at 10.7 years there is extensive T2‐hyperintensity of subcortical WM, most pronounced in fronto‐parietal WM. Diffusion is mixed with intense restriction in fronto‐orbital WM and pregenual cingulum (arrows).

**FIGURE 6:**
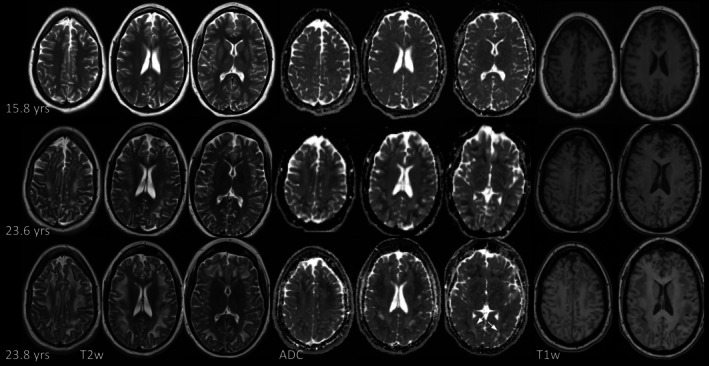
Exemplary white matter changes in p12. Except for incomplete temporopolar myelination (not depicted) WM is normal at 15.8 years. On follow‐up at 23.6 years during acute metabolic decompensation there is new T2‐hyperintensity with restricted diffusion and T1‐hypointensity. At 23.8 years, during another acute episode, T2‐hyperintense, T1‐hypointense WM changes have increased. U‐fibers are relatively spared. Diffusion remains predominantly restricted with facilitated diffusion of small areas of previously affected WM (arrows).

**FIGURE 7:**
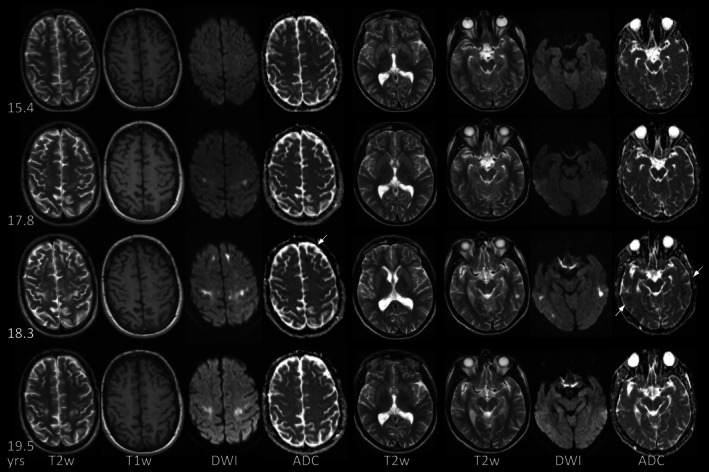
Exemplary white matter changes in p11. WM is normal in p11 at first MRI at 15.4 years. By 18 years, new, subcortical T2‐hyperintensity has developed in the bilateral precentral gyrus. At 18.3 years there is increasing involvement of frontal WM and new involvement of temporal WM with T1‐hypointensity and restricted diffusion (arrows). At 19.5 years during acute metabolic decompensation WM changes have decreased.

6/7 patients had at least one follow‐up MRI after the first appearance of subcortical WM changes. These evolved from small, uni‐ or bilateral, subcortical foci of restricted diffusion, T2/FLAIR‐hyperintensity, and variable T1‐hypointensity into bilateral, confluent, often symmetric, and sometimes extensive areas with mixed diffusion, often facilitated in areas of pre‐existing signal alteration (Figures 5, 6, 7 and S1). WM changes increased in 3/6 patients, increased and subsequently decreased in 2/6, and decreased in 1/6 with overlapping age intervals of increase and decrease. Adjacent sulci might become minimally wider or narrower with changing extent of lesions, but there was no clear space‐occupying or space‐giving effect.

Acute metabolic decompensation did not seem to be decisive for the manifestation of subcortical WM changes, as 4/10 MRI with new/increasing, but also 2/3 MRI with decreasing WM changes were acquired during acute episodes. There was no clear association with seizures, as subcortical WM changes were observed in 4/6 patients with documented seizures and in 3/7 without. Of note, WM changes seen in p5 at 11.3 and 13.8 years differed from prior cortico‐subcortical changes in young children during episodes with seizures (Figure S1 and S1) and were distinct from pre‐existing incomplete myelination.

Infratentorial WM changes were infrequent, consisting of T2‐hyperintensity of middle cerebellar peduncles and lateral medullary corpus in p7, and of central tegmental tracts in p8.

#### 3.2.6 Myelination

Overall, myelination was not adequate for age in 9/13 patients, namely in 3/5 and 6/8 first examined before and after the age of 2 years, respectively. Myelination delay normalized in p4 between 1.1 and 1.9 years, whereas mild myelin deficit confined to medial temporopolar and subinsular WM in older patients persisted in those with follow‐up.

#### 3.2.7 Volume Deficit and Thin Optic Nerve

Supratentorial volume deficit was common, seen in 9/13 patients with age at MRI 0.4–23.8 years. It manifested as widening of ventricles and sulci in 9/9, predominantly affecting dorsal cella media and frontoparietal sulci, and as a thin corpus callosum in 8/9. BCR as a surrogate parameter of supratentorial brain volume confirmed visual assessment in 8/9 patients, while in p12, basal ganglia swelling during acute decompensation resulted in “pseudo‐normalization” of inter‐caudate distance with normal BCR.

To control for decreased BCR due to swelling, we excluded MRIs acquired during acute decompensation, leaving 11 patients (27 MRI) with stable and 1 patient (3 MRI) with unknown metabolic situation. We postulated that BCR might be affected by disease duration and thus age at MRI, potentially also by cardiac involvement, as chronic heart failure is associated with lower brain volume [48]. BCR varied widely between patients, but increased with age in the five patients with follow‐up. There was no clear effect for cardiac involvement present in 4/11 patients imaged while metabolically stable (Figure S1).

Cerebellar volume deficit not restricted to watershed areas was present in five patients, all of whom also had supratentorial volume deficit.

Brainstem was thin on sagittal images in 9/13 patients imaged between 0.3 and 33.2 years. It manifested either as generalized thinning or as a relatively short and/or dorsally thin pons (Figure S1). In 6/9, brainstem was already thin at first MRI, including the youngest at 0.3 years, and in three patients on follow‐up at 0.75, 1.86, and 15.1 years. There was associated supratentorial volume deficit in 6/9 patients and generalized cerebellar volume deficit in 4/9. Vice versa, none of the four patients without thin brainstem had supratentorial volume deficit. There was no association of cerebellar watershed injury with thin brainstem: in the 2/9 patients with thin brainstem and cerebellar watershed injury, watershed injury manifested later, and 2/4 patients without thin brainstem had watershed injury.

The optic nerve was thin in four patients imaged between 2.5 and 23.8 years, namely 3 patients with optic atrophy on fundoscopy and p6 without available clinical information. MRI for visual impairment in p5 at 13.8 years without information on fundoscopy did not reveal a thin optic nerve.

#### 3.2.8 T1‐Hyperintense Pallidum and Hepatic Dysfunction

T1‐hyperintense pallidum, thought to be a hallmark of chronic hepatic insufficiency [49], was present in two patients, namely in p9 with chronic hepatic dysfunction at 18.1 years and in p7 with low cholinesterase and albumin during acute metabolic decompensation at 15.1 years (Figure S1E). None of our patients had signs of intracranial hemorrhage on conventional images or on available T2‐gradient‐echo/SWI (16/45 MRI).

## 4 Discussion

While basal ganglia involvement is a hallmark of PA and has often been the focus of imaging reports, systematic MRI analysis in our cohort of 45 MRI of 13 patients demonstrated that MR‐visible brain involvement is more widespread and complex.

Neurological manifestations in PA partly result from acute metabolic decompensation, often already at initial manifestation, but also develop with time even in patients with good metabolic control. The latter is thought to result from persistently increased levels of propionic acid, 2‐methylcitrate, lactate, and ammonia inhibiting and depleting the TCA cycle and inhibiting respiratory chain function [5]. Brain MRI in PA can therefore be expected to demonstrate

- patterns of acute neurotoxicity in patients imaged during episodes of acute metabolic decompensations, which may persist, decrease, or resolve depending on the severity of the “insult” as well as
- patterns reflecting the combined effect of chronic and (repetitive) acute neurotoxicity resulting in impaired brain maturation and/or maintenance.

### 4.1 MRI‐Sequelae of Acute Metabolic Decompensation

Deep GM involvement is a frequent imaging finding in patients with PA. It most commonly affects the basal ganglia, which has been described in 47/116 patients previously reported with MRI [12, 13, 14, 15, 17, 18, 19, 20, 21, 22, 23, 24, 26, 27, 28, 30, 31, 32, 33, 34, 35, 36, 37, 38, 39, 40, 41, 42] and 5/26 patients with either CT or MRI [25]. Deep GM changes outside the basal ganglia have been reported infrequently, affecting the thalamus in four [23, 28, 29, 32] and the dentate nucleus in two patients [50]. While dentate involvement in our patients was relatively more common (7/13), our data confirm the overall lower prevalence of deep GM involvement outside of the basal ganglia.

Basal ganglia involvement was more frequently observed in our cohort (77%) compared to the range of 0%–56% in previous cohorts [12, 16, 20, 25, 28, 36, 42]. This difference may be due to several factors: (1) In our cohort, T2‐hyperintensity of basal ganglia was often mild, e.g., isointense instead of mildly hypointense to insular ribbon, which can easily be missed. (2) Age at and timing of MRI appear to influence detection: It has been reported that basal ganglia may be normal in children prior to any severe episode beyond the neonatal period [50] and that even in patients with acute episodes beyond the neonatal period T2‐hyperintensity of basal ganglia may decrease or resolve [12, 13, 22, 23, 36, 50]. Absence of basal ganglia involvement in p3 and p6 after neonatal metabolic decompensation, in p13 and initially also p9 despite previous acute episodes beyond the neonatal phase exemplifies and confirms these observations. However, T2‐hyperintensity also resolved in p4 during adolescence despite further acute episodes. We were intrigued by the conversion of T2‐hyperintensity to T2‐ and SWI‐hypointensity between adolescence and young adulthood in p12, which might reflect increased mineralization of basal ganglia. On histopathology, vascular and parenchymal mineralization has been observed in a 35‐month‐old boy with PA [51]. We hypothesize that acute metabolic decompensation(s) in PA may accelerate the normal mineralization of basal ganglia with increasing age [52], counteracting and potentially counterbalancing T2‐hyperintensity from acute metabolic decompensation [51].

Our data confirm that, within the basal ganglia, PA preferentially affects the striatum, consisting of the putamen and caudate nucleus, and much less frequently the pallidum. The observed proportion of pallidal T2‐hyperintensity in 3/10 patients with basal ganglia involvement was similar to that of 7/27 in previously reported patients with specific mention of affected basal ganglia structures [12, 13, 15, 17, 18, 19, 20, 21, 26, 27, 30, 31, 32, 34, 35, 50]. Interestingly, temporal and diffusion patterns differed between pallidal and striatal involvement in our patients. T2‐hyperintensity of the pallidum was more intense, transient, associated with restricted diffusion, and confined to infants imaged during acute metabolic decompensation. The pallidum is a WM‐rich GM structure [53] and zones of active myelination are sensitive to energy depletion. Thus, transient T2‐hyperintensity and restricted diffusion in the imaged infants might reflect selective vulnerability during active myelination resulting in reversible vacuolization of WM. In contrast, T2‐hyperintensity of the putamen was commonly mild, long‐standing, and without regular evolution into an atrophic lesion. Movement disorder was present in 46% of our patients, which is consistent with 40% reported in the literature [1], and confined to patients with striatal involvement, including those with striatal atrophy. However, striatal T2‐hyperintensity, even if persisting for years, did not result in movement disorder in 4/6 patients, suggesting subclinical involvement.

Surprisingly, none of the 11 patients with DWI during 13 episodes of acute metabolic decompensation had restricted diffusion of the striatum. A sequence of restricted diffusion, followed by pseudonormalization and swelling in the subacute phase and by facilitated diffusion in the chronic atrophic phase, is seen not only in hypoxic–ischemic injury and ischemic stroke but also in neurometabolic diseases with so‐called metabolic stroke, e.g., affecting the striatum during acute encephalopathic crises in glutaric aciduria type 1 (GA1) [54] or pallidum in methylmalonic acidemia (MMA) [55]. Metabolic stroke, namely the acute onset of central neurological deficit in the absence of vessel occlusion or rupture and a pattern not consistent with vascular territories, is seen in inborn errors of metabolism including mitochondrial diseases, urea cycle defects, and organic acidurias [56]. It has also been reported for PA, often, but not necessarily, associated with acute metabolic decompensation. The latter suggests isolated/focal metabolic decompensation in the CNS and CSF without “peripheral” metabolic acidosis and hyperammonemia [3, 22]. Clinical findings in eight reported patients with potential metabolic stroke [13, 17, 21, 22, 23, 30, 39, 40] encompass altered mental state, unilateral weakness, seizures, aphasia, and Parkinsonian syndrome, while reported imaging findings include basal ganglia and cortico‐subcortical changes, atrophy, and normal findings. Restricted diffusion with low ADC of putamen and cerebellum has been reported for one case [21], transient hyperintensity of basal ganglia and cortex on T2‐ and diffusion‐weighted images for another (ADC not mentioned) [22]. In our cohort, three patients with acute metabolic decompensation, evolving or new movement disorder, and acute onset of muscular hypotonia and seizures in 2/3 were clinically classified as suffering “metabolic stroke” (p1, p2, p5). MRI demonstrated striatal T2‐hyperintensity in all, cortico‐subcortical involvement in the two patients with seizures, and in the two infants, additional transient T2‐hyperintensity and restricted diffusion of pallidum. Thus, clinical and MRI findings of acute decompensation in PA appear to be quite diverse [30], in particular compared to the lesional pattern with initially restricted diffusion in GA1 and MMA.

There is currently no conclusive hypothesis that adequately explains the different patterns of basal ganglia involvement. The main biochemical difference between PA and MMA is the increase of methylmalonate in MMA. In addition to the effects due to propionyl‐CoA and 2‐methylcitrate common to both, PA and MMA [57], methylmalonate inhibits succinate transport, blocking an important compensatory anaplerotic mechanism for TCA problems. Moreover, methylmalonate, as a dicarboxylic acid, cannot freely cross the blood–brain barrier, resulting in intracerebral trapping of endogenous methylmalonate with potential aggravation [58]. In GA1, glutarate and 3‐OH‐glutarate are also trapped and interfere with succinate transport [57]. The lower frequency of lesion development in PA might thus be related to less severe inhibition of TCA.

Involvement of supratentorial cortex and cerebellar watershed areas was also associated with acute neurotoxicity. Supratentorial cortex was more frequently affected when acute metabolic decompensation was accompanied by seizures/status epilepticus. This is consistent with previously reported patients [19, 22, 23, 24, 29, 30, 33, 38], as is concurrent involvement of subcortical WM and resolution and/or recurrence in different locations. An apparent age predilection for cortical involvement in younger patients of our group likely resulted from the higher proportion of younger patients with seizures during decompensation (4/5 and 2/9 imaged before and after 5 years, respectively). In patients with seizures, we cannot exclude that MRI changes were at least partially related to seizure activity. Moreover, as ammonia is elevated during acute metabolic decompensation, hyperammonemia may be an additional contributing factor. However, the pattern of cortex involvement in our patients was not characteristic of acute hepatic encephalopathy, which typically involves insular or cingulate cortex and/or cortex with changes preferentially affecting the depth of sulci [59, 60].

Cerebellar watershed injury has a characteristic pattern, affecting cerebellar GM and directly adjacent WM at the depths of cerebellar fissures in the border zone between superior, anterior, and inferior cerebellar artery, usually bilaterally [61]. It has been reported in children with hypoxic–ischemic injury or less commonly with posterior reversible encephalopathy syndrome [61], with non‐accidental head injury [62], during acute, severe manifestations of inherited monoamine neurotransmitter disorders, particularly dihydropteridine reductase deficiency [63], and encephalopathic episodes or prolonged status epilepticus in *leucine*‐tRNA synthetase 1 (LARS1) [64]. A similar pattern is depicted in a 4‐year‐old PA patient with regression after prolonged gastro‐intestinal infection [21] and was observed in 4/13 patients of our group. As far as extrapolating from a small number of MRIs is possible, seizures during acute episodes of PA apparently predispose one to acute cerebellar watershed injury.

### 4.2 Brain Maturation and Maintenance: Myelination, Volume Deficit, and a Subcortical Leukoencephalopathy

Chronic neurotoxicity may interfere with normal brain maturation and maintenance depending on the extent of exposure in the form of disease duration and metabolic control, potentially aggravated by episodes of acute metabolic decompensation.

Delayed or incomplete myelination is a common, non‐specific manifestation of impaired brain maturation in inherited and acquired diseases. It has been observed in previously reported individuals with PA [12, 15, 20, 28], was common in our group and mild.

Volume deficit is a similarly common finding, and predominantly supratentorial volume atrophy has been observed in previously reported patients. Using *z*‐scores of BCR as a quantitative, age‐ and sex‐controlled surrogate parameter of supratentorial brain volume to delineate temporal changes, variation between patients of similar age was large. This is likely due to differing numbers and severity of acute episodes, residual enzyme activity, and metabolic control, which, however, we could not separately address with the available information. At an individual level, however, BCR as an indicator of supratentorial volume deficit increased over time in patients with follow‐up, consistent with an effect of chronic neurotoxicity.

An unexpectedly common finding in our patients was a thin brainstem with abnormal brainstem contour on sagittal images with generalized thinning, a relatively thin pons with a variable “dorsally excavated” aspect, and relatively short rostro‐caudal pons. Its aspect was similar to patients with LARS1‐deficiency [64]. There was no clear temporal pattern, as the brainstem was already thin at first MRI in our youngest patient at 0.3 years and became thin only on follow‐up in 3/9 patients, which might reflect a combination of hypoplasia and volume loss. One previous publication has reported pontine hypoplasia in 1/8 patients [33].

WM changes in PA have previously been reported without further specification for 2/8 and 6/16 patients [33, 36], in centrum semiovale, subcortical temporal and insular WM in a 16‐year‐old patient with a psychotic episode [26], and as diffuse hyperintensity of subcortical WM on DWI in a 5‐month‐old child during acute metabolic decompensation [29]. In our cohort, a pattern of predominantly subcortical WM changes becoming more frequent with age was observed in older children and young adults, occurring in more than 50% of all patients and in more than 75% of patients examined after the age of 5 years. Small foci of restricted diffusion, T2/FLAIR‐hyperintensity, and variable T1‐hypointensity evolved into bilateral, sometimes extensive changes with restricted and facilitated diffusion, centripetal extension, decreasing during adolescence and young adulthood in 3/6 with follow‐up. Restricted diffusion in WM diseases is typically seen in disorders with myelin microvacuolation. These include diseases directly affecting ion channels and gap junctions involved in brain water homeostasis, but also neurometabolic diseases like Canavan's disease, maple syrup urine disease, non‐ketotic hyperglycinemia, and some mitochondrial leukodystrophies [65, 66]. Normally, potassium released on neural activity under and within the myelin sheath together with osmotically obliged water is rapidly siphoned and dispersed via the panglial syncytium. It uses gap junctions coupling myelin lamellae and linking the outermost myelin lamella with the extensive astrocyte network and is driven by electrical and osmotic gradients, preventing intramyelinic edema [67]. For PA, inhibition of Na+/K+‐ATPase by propionate, inhibition of the TCA cycle, and depletion of TCA cycle intermediaries might interfere with the normal long‐distance disposal of water and potassium by the panglial syncytium. Resulting myelin microvacuolation may persist, deteriorate to macrovacuolation with facilitated diffusion, and/or resolve. This might explain the presence of restricted and facilitated diffusion and the potential for resolution.

It is unclear why some leukodystrophies have a subcortical predominance, a relatively infrequent pattern with (currently) a limited differential diagnosis. WM changes in PA, which primarily involve GM, do not fulfill the definition of leukodystrophies as genetically determined disorders of any WM component with selective and primary involvement of CNS WM [68] and fall under the broader term of leukoencephalopathy [65]. The characteristic subcortical WM changes in PA, however, tend to appear later than deep GM involvement, may be the dominant imaging feature in older children and young adults and thus mimic WM diseases, in particular those with concurrent deep gray matter involvement, e.g., mitochondrial leukodystrophies, L‐2‐hydroxyglutaric aciduria, maple syrup urine disease, or Canavan's disease [65]. We therefore believe that it is important to consider PA in the differential diagnosis of WM diseases with predominantly subcortical involvement, namely L‐2‐hydroxyglutaric aciduria, Canavan's disease, Kearns–Sayre syndrome, and leukencephalopathy with thalamus and brainstem involvement and elevated lactate [65].

Our study is limited by its retrospective, observational nature, with local clinical assessments mirroring the individuals' situation instead of prospective clinical and imaging assessments at regular time points. MRI studies are non‐uniform regarding acquisition, age at MRI, indication, and number of follow‐up scans. Statistical evaluation was thus only explorative and descriptive. Our deductions regarding disease progression and histopathological correlates remain hypotheses that need to be validated by future, prospective studies where (more) standardized MRI may allow additional, observer‐independent MRI analysis using statistical learning; integrating MR spectroscopy might offer insight into accumulating neurotoxic metabolites. Nevertheless, the current study presents the largest cohort in terms of the number of systematically analyzed MRI studies in individuals with PA.

To conclude, PA is a GM disease preferentially involving the striatum with mild, often prolonged T2‐hyperintensity, the potential to normalize, and relatively infrequent lesion development. Transient T2‐hyperintensity and restricted diffusion of the pallidum in infants during acute metabolic decompensation might reflect the selective vulnerability of WM‐rich pallidum during active myelination. Cerebellar watershed injury is associated with acute metabolic decompensation complicated by seizures and—as in other neurometabolic diseases—may indicate severe decompensation. A characteristic pattern of predominantly subcortical WM changes with initially restricted diffusion is a frequent finding in older children and young adults with PA, likely reflecting myelin vacuolization.

## Author Contributions

F.H. and I.H. had the idea for the study, and H.F.‐P., F.H., and I.H. designed the study. S.K. designed and coordinated the E‐IMD observational study. All authors examined patients and/or collected data. All MRIs were evaluated by H.F.‐P. and I.H. H.F.‐P., F.H., and I.H. drafted the article. I.H. created all figures. All authors revised the manuscript critically for important intellectual content and approved the submission.

## Ethics Statement

All procedures were in accordance with the ethical standards of the responsible committee on human experimentation (institutional and national) and with the Helsinki Declaration of 1975, as revised in 2024. Ethics approval was obtained from the institutional ethics committee of the Medical Faculty, University of Heidelberg (S‐525/2010).

## Consent

Informed consent was obtained locally from all individuals and their parents in the case of minors.

## Conflicts of Interest

The authors declare no conflicts of interest.

## Supporting information


**Data S1:** jimd70101‐sup‐0001‐Supinfo.pdf.

## Data Availability

The MRI images are not publicly available under data protection laws.
