# Supplementary material for: Propionic Acidemia: Gray Matter Disease Meets Subcortical Leukodystrophy
Source: J Inherit Metab Dis. 2025 Nov 4;48(6):e70101. doi: 10.1002/jimd.70101 (PMC12587030; doi:10.1002/jimd.70101)
Supplement: Supplementary file 1 — Data S1: jimd70101‐sup‐0001‐Supinfo.pdf. [file JIMD-48-0-s001.pdf]

Supplementary Material for Fels-Palesandro, Hörster et al.
"Propionic acidemia: grey matter disease meets subcortical leukodystrophy."

Supplementary Table 1: Overview of patients, diagnosis, and clinical findings.

Supplementary Figure 1: Striatal atrophy and T1-hyperintense pallidum.

Supplementary Figure 2: Evolving subcortical white matter changes in p5.

Supplementary Figure 3: Plots of age- and sex-normalized z-scores of bicaudate ratio (BCR).

Supplementary Figure 4: Thin brainstem on midsagittal images.

Supplementary Table 1: Overview of patients, diagnosis, and clinical findings.

	onset		diagnosis		death	MRI		clinical findings at time of MRI						NH3, glycine in serum [µmol/l]		
PID	type	age [d]	mode	genetic variants (PCCA/PCCB: p-code, c-code, if/as available)	[yrs]	FU	age [yrs]	neuology	mov. disorder	optic atrophy*	cardiac involv.	chronic kidney disease	chronic hepatic dysfct.	NH3 at MRI	max. NH3 of episode before MRI	glycine: max. of episode before MRI (max)/ at MRI (MRI) [age-dependent normal values]
p1	NA	112	NBS	PCCB: p.(Met1?) c.3G>C p.(His258Pro), c.773A>C		FU0	0,3	somnolence, musc. hypotonia	n	unkn.	n	n	n	368	452	811 (max) [60-380]
						FU1	0,3	coma, severe musc. hypotonia, dystonia	y	unkn.	n	n	n	86	452	108 (MRI) [60-380]
p2	EO	2	select.	PCCB: p.Arg 113*, c.337C>T		FU0	0,4	new seizures, musc. hypotonia	n	unkn.	n	unkn.	unkn.	170	170	unkn.
						FU1	0,7	dyskinesia, musc. hypotonia, delayed motor dev.	y	unkn.	n	n	n	70		526 (MRI) [60-380]
p3	EO	4	select.	PCCA: p.Thr168fs, Thr168fs	17	FU0	1,1	dev. delay	n	unkn.	n	n	n	72		450 (MRI) [60-380]
p4	EO	3	select.	PCCB: homozygous deletion exon 1-7		FU0	1,1	focal seizures, mild truncal hypotonia	n	amblyopia (left eye)	n	n	n	76	122	1582 (max) [60-380]
						FU1	1,9	irritability, seizures, truncal hypotonia, right-sided hyperreflexia, musc. hypertonia, loss	n	amblyopia (left eye)	n	n	n	80	180	342 (MRI) [60-380]
						FU2	1,9	unchanged	n	amblyopia (left eye)	n	n	n	62		1108 (max)[60-380]
						FU3	12,1	impaired consciousness, seizure, hyponatremia, thr.cytopenia, musc.	n	amblyopia (left eye)	CMP	n	n	69	131	207 (MRI) [110-343]
						FU4	12,6	musc. hypotonia, global dev. delay, chron. otitis media (l), clin. susp. cholesteatoma	n	amblyopia (left eye)	CMP	unkn.	unkn.	36		1493 (MRI) [110-343]
						FU5	13,2	new nystagmus, musc. hypotonia, global dev. delay	n	amblyopia (left eye)	CMP	n	n	71	150	1192(MRI) [110-343]
p5	EO	2	select.	PCCB: p.G407fs/p.G112S		FU0	1,2	complex focal seizures, pre-existing truncal hypotonia, mild ataxia	n	unkn.	n	n	n	unkn.		692 (max) [60-380]
						FU1	2,8	non-convulsive status epilepticus, truncal hypotonia, dystonia, dysarthria	y	unkn.	n	n	n	51	230	168 (MRI) [60-360]
						FU2	3,0	no details	unkn.	unkn.	n	n	n	99		1609 (MRI) [60-380]
						FU3	3,4	vomiting after fall on head, no details	unkn.	unkn.	n	n	n	92		1015 (MRI)[110-343]
						FU4	11,3	seizures, 2 month earlier slight dysarthria	unkn.	unkn.	n	n	n	unkn.		unkn.
						FU5	13,8	no details; at 12.8 yrs dystonia, scoliosis, truncal hypotonia	y	unkn.	n	n	n	unkn.		unkn.
p6	EO	4	select.	unknown	15	FU0	2,5	mental retardation	n	unkn.	n	n	n	unkn.		unkn.
p7	EO	1	fam.	PCCB: p.[Arg512Cys, Arg512Cys]	18	FU0	3,6	seizures, dev. delay (speech > motor), athetoid movements (extremities)	y	unkn.	n	n	n	47		554 (MRI), elevated
						FU1	15,1	mus. hypotonia, dystonia, conductive hearing loss, intell. disability	y	y	CMP	n	(low cholin-esterase, albumin, hepatomeg.)	120	120	1221 (max), 566 (MRI) [147-299]

Supplementary Table 1: Overview of patients, diagnosis, and clinical findings (continued; legend next page).

	onset		diagnosis		death	MRI		clinical findings at time of MRI						NH3, glycine in serum [µmol/l]		
PID	type	age [d]	mode	genetic variants	[yrs]	FU	age [yrs]	neuology	mov. disorder	optic atrophy*	cardiac involv.	chronic kidney disease	chronic hepatic dysfct.	NH3 at MRI	max. NH3 of episode before MRI	glycine: max. of episode before MRI (max)/ at MRI (MRI) [age-dependent normal values]
p8	NA	154	NBS	PCCB: p.[Arg113*, Ser207Phe]		FU0	5,6	dysarthria, severe dystonia of arms and feet, musc. hypotonia, intell. disability	y	unkn.	n	n	n	69		not investigated
						FU1	8,1	first complicated seizure, otherwise stable	y	unkn.	n	n	unkn.	62		1799 (MRI) [110-343]
						FU2	8,4	pre-operativ MRI	y	unkn.	n	n	unkn.	33		1479 (MRI) [110-343]
						FU3	8,7	seizures resolved, otherwise unchanged	y	unkn.	long QTc	n	n	66		1485 (MRI) [110-343]
						FU4	9,2	unchanged	y	unkn.	long QTc	n	unkn.	52		not investigated
						FU5	10,0	unchanged	y	unkn.	long QTc	n	unkn.	17		1354 (MRI) [110-343]
						FU6	11,7	unchanged	y	unkn.	long QTc	n	unkn.	unkn.		1681 (MRI) [110-343]
						FU7	13,5	unchanged	y	y	long QTc	n	n	38		1587 (MRI) [110-343]
						FU8	14,7	unchanged	y	y	long QTc	n	unkn.	unkn.		1414 (MRI) [110-343]
p9	EO	7	select.	PCCB: p.[Arg26fs, Arg376Leu]		FU0	9,0	follow-up (no details)	unkn.	unkn.	left ventr. hypertr.	n	n	74		1546 (MRI) [60-380]
						FU1	11,6	alternating impaired consciousness,	n	n	left ventr. hypertr.	n	n	97	129	453 (MRI) [110-343]
						FU2	18,1	new tremor, intell. disability	n	n	left ventr. hypertr.	CRF stage 3A	y	50	120	1083 (MRI, max) [110-343]
p10	EO	7	select.	PCCB: p.[Arg512Cys, Arg512Cys]	19	FU0	9,8	status epilepticus, somnolence, intell. disability	unkn.	n	long QTc	n	unkn.	126	174	409 (max) [110-343]
						FU1	10,7	musc. hypertonia, hyperreflexia, and cloni o lower extr., progressive gait disturbance with drop foot; sensorineural hearing loss, optic atrophy	y	n	long QTc	n	unkn.	78		1327 (MRI) [110-343]
p11	EO	7	select.	unknown	20	FU0	15,4	intell. disability, musc. hypotonia	n	unkn.	long QTc	unkn.	unkn.	unkn.		unkn.
						FU1	18,0	intell. disability (IQ 53)	n	unkn.	long QTc	unkn.	unkn.	unkn.		unkn.
						FU2	18,3	no details	unkn.	unkn.	long QTc	unkn.	n	unkn.		unkn.
						FU3	19,5	no details	unkn.	unkn.	long QTc, CMP	unkn.	n	unkn.	unkn.	unkn.
p12	EO	28	select.	unknown		FU0	15,8	new optic atrophy, impaired fine motor skills, intell. disability	n	y	n	n	n	100		949 (MRI) [100-384]
						FU1	23,6	intell. disability, psychosis	n	y	long QTc	n	steatosis hepatis not related to PA	95	178	1106 (MRI) [100-384]
						FU2	23,8	intell. disability, psychosis	n	y	long QTc	CRF stage 2	steatosis hepatis not related to PA	109	124	837 (MRI) [100-384]
p13	EO	11	select.	unknown		FU0	17,7	fatigue, vomiting, intell. disability	n	n	long QTc	n	n	36		1215 (max), 1146 (MRI)
						FU1	22,9	psychosis, acute hallucination, intell. disability	n	n	long QTc	n	n	62		1233 (max) [100-384]
						FU2	23,5	fatigue, vomiting, intell. disability	n	n	long QTc	n	n	57		1084 (max) [100-384]
						FU3	33,2	sensorineural hearing loss, intell. disability	n	n	long QTc	n	unkn.	unkn.		not investigated

Legend for Suppl. Table 1:Overview of patients, diagnosis, and clinical findings.
CMP: cardiomyopathy; CRF: chronic renal failure; EO/LO: early/late onset before/after 28 days; decomp.: decompensation; dev.: development; fam.: high risk family screening, FU: follow-up MRI; mo: months; mov.: movements; musc.: muscular; NBS: newborn screening; progr.: progressive; select: selective screening; yrs: years; *unknown if no fundoscopy performed.

Supplementary Figure 1: Striatal atrophy and T1-hyperintense pallidum.

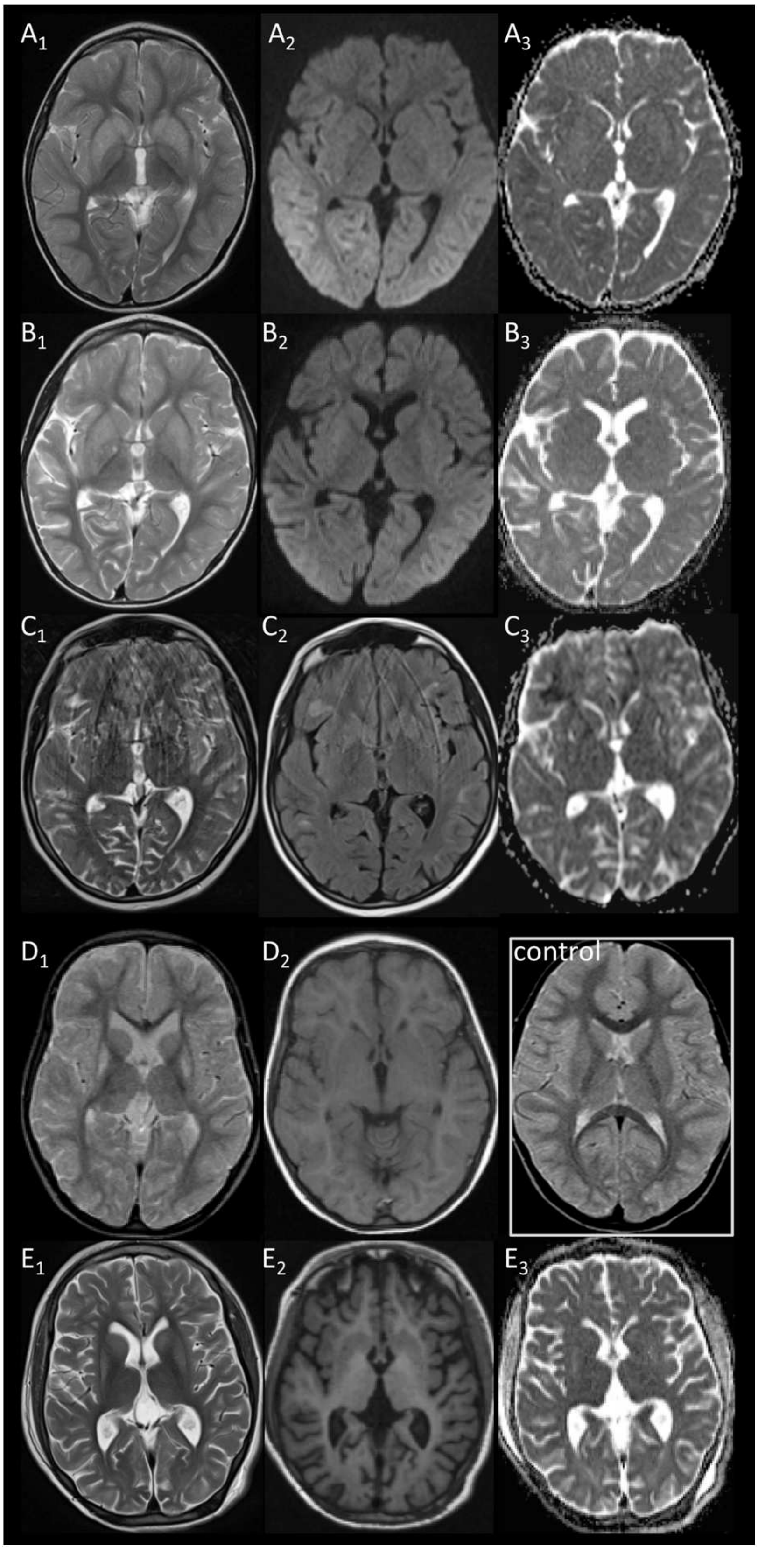

(A-C) Mottled T2-hyperintensity, swelling, and facilitated diffusion of putamen and caudate on day 4 of acute metabolic decompensation with complex focal seizures in p5 at 2.8 years (FU1; A). NB cortico-subcortical swelling and restricted diffusion in right parieto-occipital and frontal lobes.
(B) On follow-up 1.7 months later striatum is less T2-hyperintense and diffusion has normalized. NB right parieto-occipital atrophy and facilitated diffusion.
(C) On MRI at for seizures at 11.3 years (FU4), striatum is still T2/FLAIR-hyperintense, but has become atrophic with widened perivascular spaces. NB new, subcortical WM changes with restricted diffusion and without cortical involvement (see also suppl.Fig. 2).

(D, E) First MRI in p7 at 3.6 years for seizures, developmental delay and athetoid movements of arms and legs demonstrates T2-hyperintense, already atrophic putamen (D_1, compare with control). Pallidum is normal on T1w images (D_2). At 15.1 years (E), on the day of admission for acute metabolic decompensation, five days after upper respiratory tract infection, the caudate is newly hyperintense, though less than the atrophic putamen, and diffusion is facilitated (E_3). Pallidum has become T1-hyperintense (E_2).

(T2w: A-E_1; DWI: A-C_2; ADC-maps: A-C_3, E_3; T1w: D,E_2)

Supplementary Figure 2: Evolving subcortical white matter changes in p5.

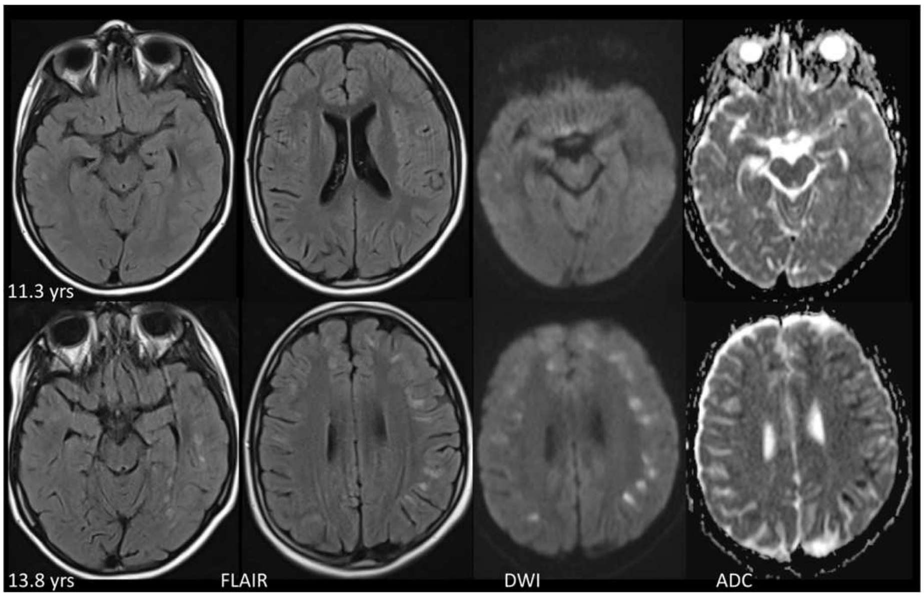

Focal, subcortical WM changes in p5 with punctate FLAIR-hyperintensities in right temporal and frontal lobes with restricted diffusion at 11.3 years.

Increasing, multiple, foci with predominantly restricted diffusion affecting all lobes on follow-up at 13.8 years.

Supplementary Figure 3: Plots of age- and sex-normalized z-scores of bicaudate ratio (BCR).

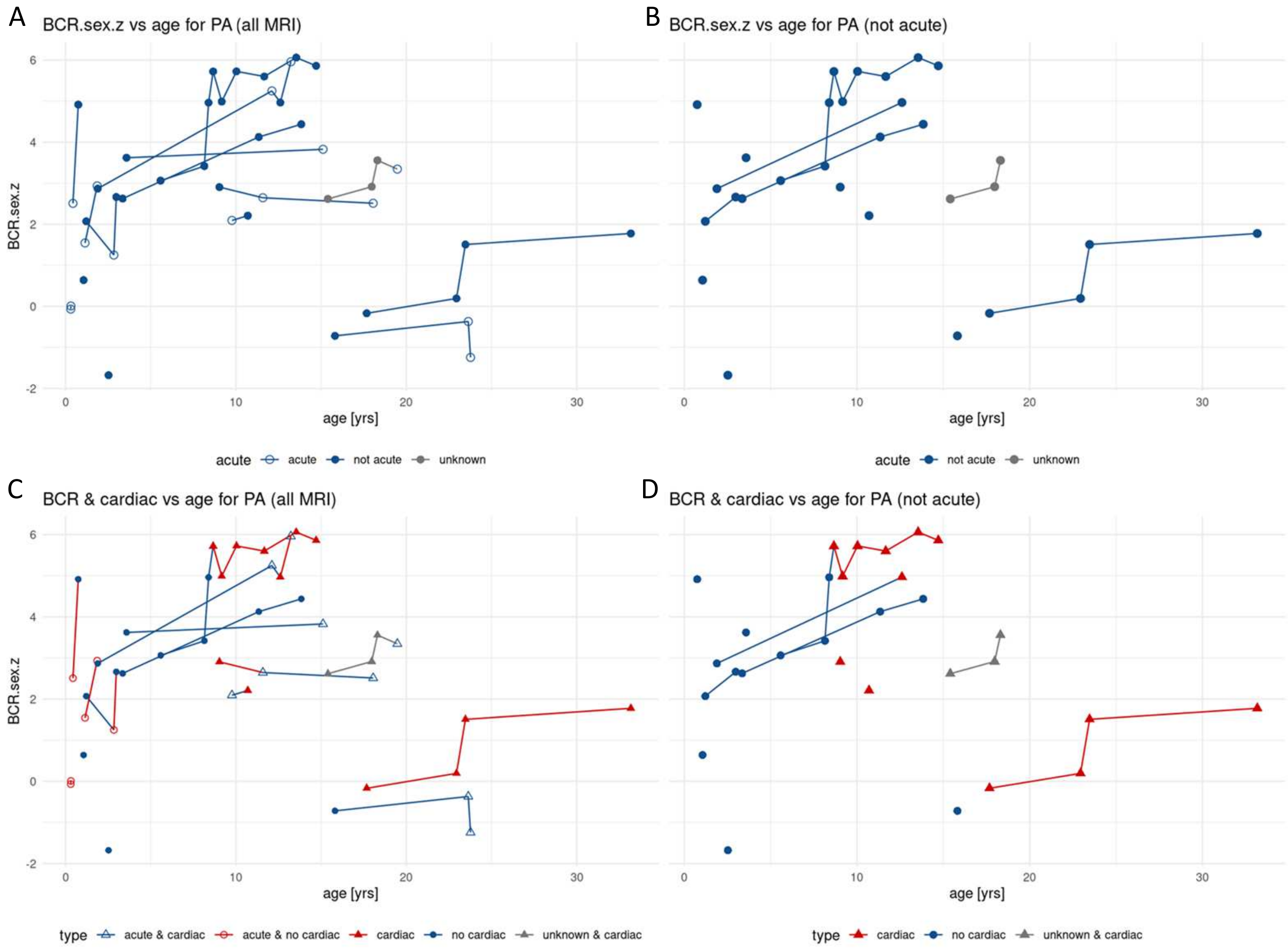

(A) Plots for all MRI versus age and (B) only for MRI not performed for acute metabolic deceompensation (lines connect values from multiple MRI) show wide variation of BCR between patients and an intraindividual increase with age.
(C) Plot with colour-coding for cardiac involvement for all MRI and (D) only for MRI not performed for acute episodes without clear effect for cardiac involvement on BCR.

Supplementary Figure 4: Thin brainstem on midsagittal images.

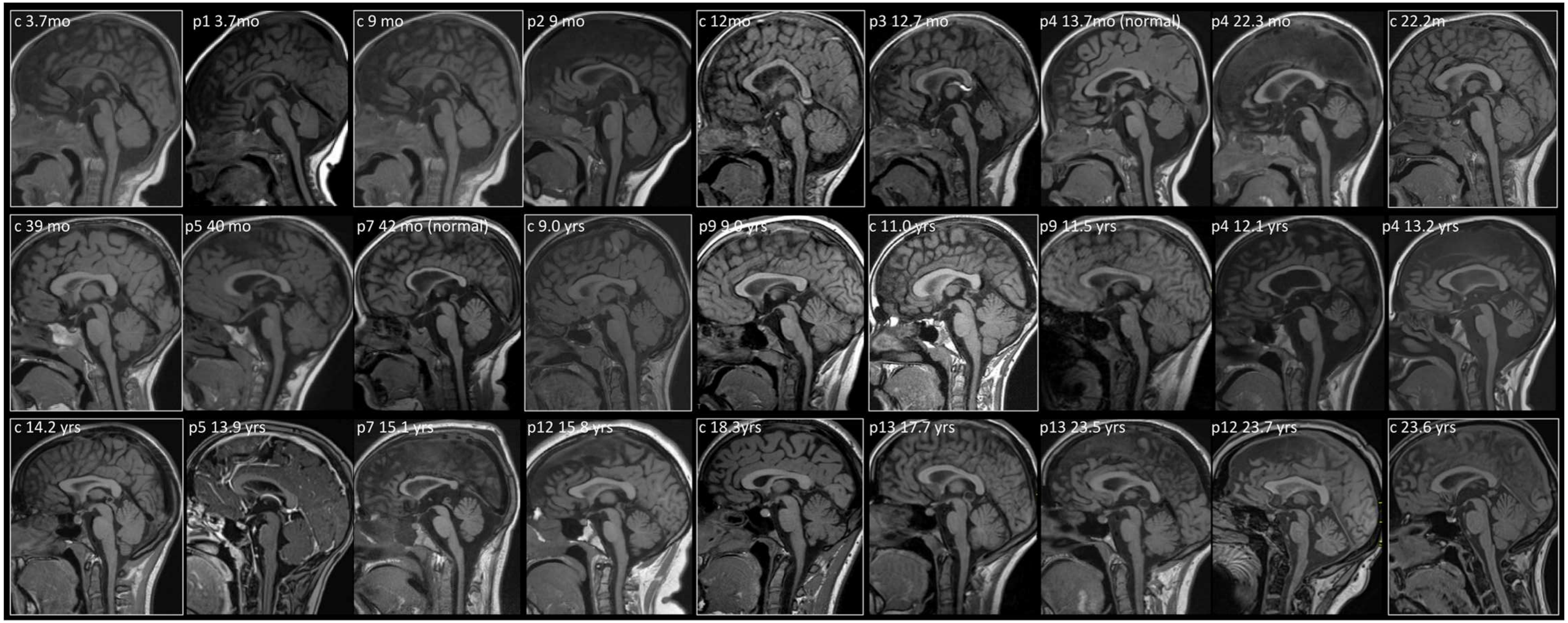

Images depicting generalized thin brainstem or a relatively short and/or dorsally thin pons are sorted in order of increasing age at MRI. Age in years and PID are denoted in upper right corner, control images by “c”.
